# Thermal response test data of five quadratic cross section precast pile heat exchangers

**DOI:** 10.1016/j.dib.2018.02.080

**Published:** 2018-03-08

**Authors:** Maria Alberdi-Pagola

**Affiliations:** Department of Civil Engineering, Aalborg University, Denmark

## Abstract

This data article comprises records from five Thermal Response Tests (TRT) of quadratic cross section pile heat exchangers. Pile heat exchangers, typically referred to as energy piles, consist of traditional foundation piles with embedded heat exchanger pipes. The data presented in this article are related to the research article entitled *“Comparing heat flow models for interpretation of precast quadratic pile heat exchanger thermal response tests”* (Alberdi-Pagola et al., 2018) [1]. The TRT data consists of measured inlet and outlet temperatures, fluid flow and injected heat rate recorded every 10 min. The field dataset is made available to enable model verification studies.

**Specifications Table**

**Table:** 

Subject area	Engineering, Renewable energies
More specific subject area	Shallow geothermal energy applications and soil investigation techniques.
Type of data	Tables in Excel sheets.
How data was acquired	The field data was acquired with a Kamstrup Multical ® 801.
Data format	Raw.
Experimental factors	Five tests were performed in different pile heat exchangers, i.e., different length and pipe configurations.
Experimental features	During the TRT, the heat carrier fluid (water) is circulated in the ground heat exchanger while being continuously heated at a specified rate. Heat dissipates to the ground heat exchanger and subsequently to the ground. The test records fluid inlet- and outlet temperatures, the fluid flow rate and energy consumption and logs them in 10-min intervals for at least 48 h. The tables also provide the accumulated energy and volume.
Data source location	The data analysed have been collected in two different locations in Denmark:
	Langmarksvej test site in Horsens (55° 51′ 43″ N, 9° 51′ 7″ E), where energy piles LM1, LM2 and LM3 have been tested.
	Rosborg test site in Vejle (55° 42′ 30″ N, 9° 32′ 0″ E), where energy piles RN1 and RS1 have been tested.
Data accessibility	The data are available with this article.

**Value of the data**

• Each TRT is presented in an individual Excel sheet.
• These data can be used to validate thermal models of pile heat exchangers.
• There are not publicly available full-scale TRT datasets for pile heat exchangers.
• Sharing data will support the development of this type of ground heat exchangers.
• These data can supplement other data sets to assist the development of thermal dimensioning guidelines for pile heat exchanger foundations.

## 1 Data

**Fig. 1:**
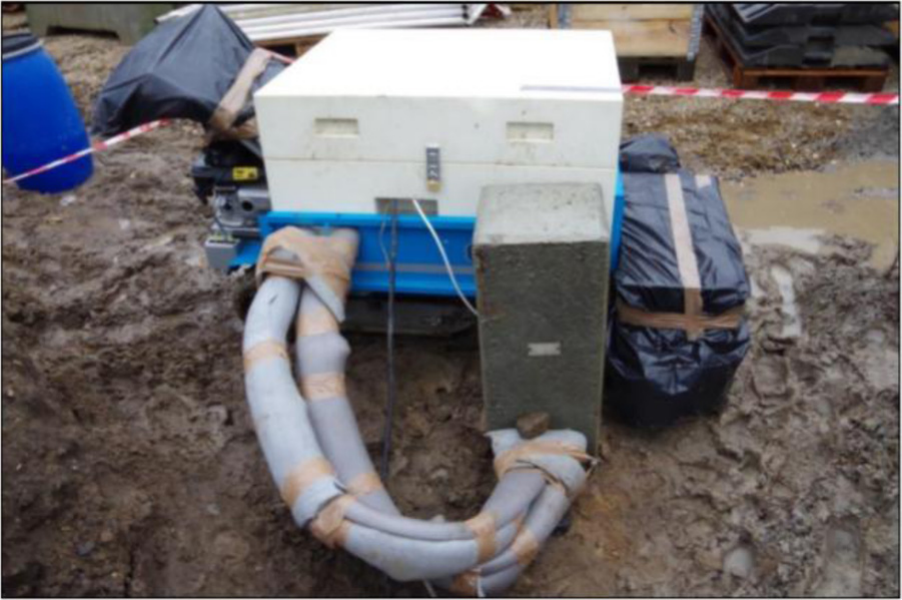
Ongoing TRT at Langmarksvej. Inlet- and outlet pipes are insulated to prevent disturbances from ambient temperature conditions [2].

Dimensioning of Ground Source Heat Pump installations typically relies on thermal response testing (TRT) of one or more ground heat exchangers. The dataset of this article provides raw TRT data of several precast pile heat exchangers, described in [1]. Fig. 1 shows the setup for one of the tests.

## 2 Experimental design, materials and methods

During the TRT, the heat carrier fluid (water) is circulated in the ground heat exchanger while being continuously heated at a specified rate. Heat dissipates to the ground heat exchanger and subsequently to the ground. The test records fluid inlet- and outlet temperatures, the fluid flow rate and energy consumption and logs them in 10-min intervals for at least 48 h.

The shared data consists of five TRTs of square cross section precast pile heat exchangers. The five energy piles have different lengths and pipe configurations. The tests have been carried out at two different locations in Denmark.

Model interpretation of the measured TRT temperatures yield estimates of the undisturbed soil temperature T_0_ [°C], the average soil thermal conductivity λ_s_ [W/m/K] over the length of the heat exchanger and the thermal resistance the ground heat exchanger R_b_ [K m/W] [3], [4], [5].

The TRT sets are compiled in a single Excel file, separated in sheets named by the pile IDs (refer to Table 2 in [1]). Each sheet is divided in seven columns, namely: date, accumulated heat energy [kWh], accumulated volume [m^3^], inlet temperature T1 [°C], outlet temperature T2 [°C], flow [l/h] and injection heat rate or effect [kW]. Notice the data is given from the closest in time to the most distant.

The TRT equipment is produced by UBeG, Ref. [6]. The temperature sensors are Pt 500 and Pt 1000 type and the flow-meter is an ultrasonic flowmeter Ultraflow® type by Kamstrup. The records are compiled by a Kamstrup Multical 801 logger. The equipment is further described in Ref. [2].
