# Supplementary material for: Thermal response test data of five quadratic cross section precast pile heat exchangers
Source: Data Brief. 2018 Mar 8;18:13–5. doi: 10.1016/j.dib.2018.02.080 (PMC5996141; doi:10.1016/j.dib.2018.02.080)
Supplement: Supplementary file 1 — Transparency document. [file mmc1.docx]

Declarations of interest: none.
